# Supplementary material for: EP-PINNs: Cardiac Electrophysiology Characterisation Using Physics-Informed Neural Networks
Source: Front Cardiovasc Med. 2022 Feb 3;8:768419. doi: 10.3389/fcvm.2021.768419 (PMC8850959; doi:10.3389/fcvm.2021.768419)
Supplement: Supplementary file 1 [file Data_Sheet_1.PDF]

Supplementary Tables

Parameter	Value Used	Additional Values
**a**	0.010	0.002
**b**	0.150	0.075
**ε**	0.002	-
**k**	8.00	-
**μ_1**	0.20	-
**μ_2**	0.30	-
**D**	0.10 mm^2/TU	0.02 mm^2/TU

Supplementary Table 1 - Values used for model parameters in the monodomain Aliev Panfilov model, from (Göktepe and Kuhl, 2009; Nash and Panfilov, 2004)*. All variables are in consistent arbitrary units (AU), except D. TU: time units. The values in the 3rd column were used in additional tests to test EP-PINNs performance with different parameters.*

	EP-PINNs Arquitecture	Training Scheme	Typical Training Time	Train, Test Data Split (%, #points)
**1D**	A: 4 layers x 32 neurons	1	25 min	10% ($1.40x10^2$ pts), 90% ($1.26x10^3$ pts)
**2D Homogeneous**				
Planar or Centrifugal Wave	A: 4 layers x 32 neurons	2<br>$iter_1$ = 1.5 x 10^4; lr_2 = 5 x 10^{-4} ; $iter_2$ = 1.2 x 10^5	2.5 h	20% ($1.40x10^5$ pts), 80% ($5.60x10^5$ pts)
Spiral Wave	A: 5 layers x 60 neurons	2<br>$iter_1$ = 1.5 x 10^4; lr_2 = 10^{-4}; $iter_2$ = 1.5 x 10^5	7 h	
**2D Heterogeneous**				
Centrifugal Wave	B: 4 layers x 32 neurons	2<br>$iter_1$ = 1.5 x 10^4; lr_2 = 5 x 10^{-4}; $iter_2$ = 1.2 x 10^5	7 h	20% ($1.40x10^5$ pts), 80% ($5.60x10^5$ pts)
Spiral Wave	B: 5 layers x 60 neurons	2<br>$iter_1$ = 1.5 x 10^4; lr_2 = 10^{-4}; $iter_2$ = 1.5 x 10^5	16 h	
**Canine Atrial Model**	4 layers x 32 neurons	1	15 min	10% ($1.40x10^3$ pts), 90% ($1.26x10^3$ pts)
**Optical Mapping Data**	A: 6 layers x 60 neurons	2<br>$iter_1$ = 5 x 10^3; lr_2 = 10^{-5}; $iter_2$ = 1.5 x 10^5	15 min	80% (116-200 pts), 20% (29-50 pts)

Supplementary Table 2 – Details of the network architecture, training schemes and typical training times used in the applications listed in this study. Details of architectures A and B and training schemes 1 and 2 can be found in Figure 2. The same settings were used for forward and inverse modes of EP-PINNs. In architecture B, the same number of layers and neurons is used in NN and NN_D.

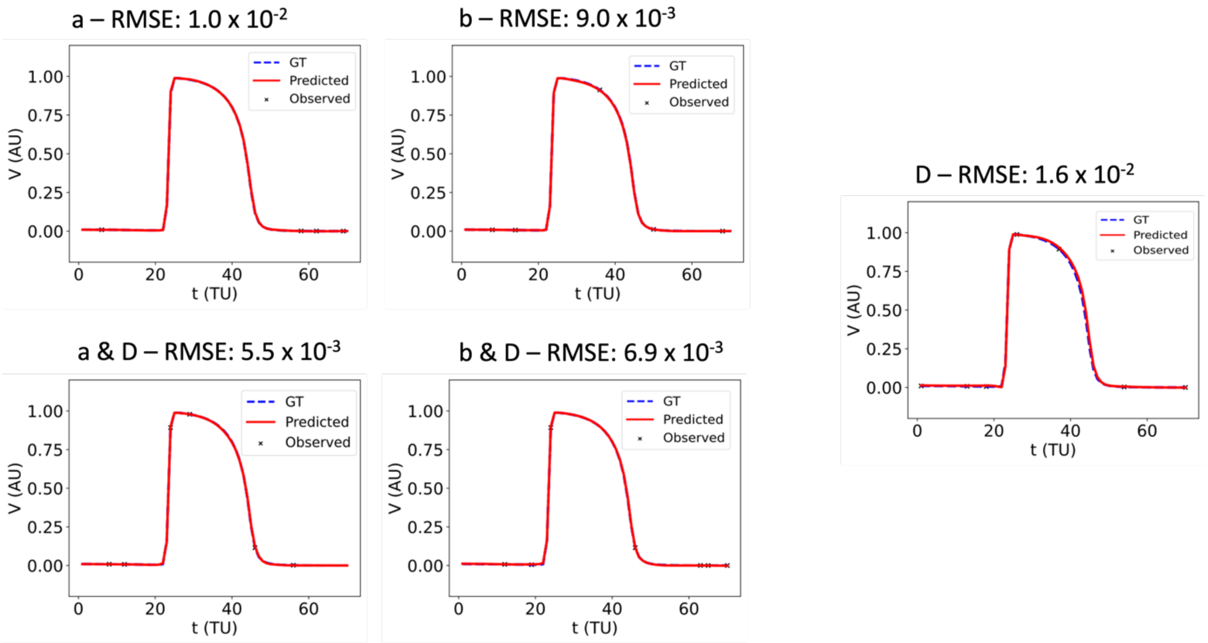

Supplementary Figure 1 – Representative action potentials in the 1D inverse scenario, for estimates of parameters: a (top left), b (top right), a & D (bottom left), b & D (bottom right) and D (far right). The total RMSE for V estimates (in AU) for each case is also shown.

References

Göktepe, S., and Kuhl, E. (2009). Electromechanics of the heart: a unified approach to the strongly coupled excitation–contraction problem. *Comput. Mech.* 45, 227–243. doi:10.1007/s00466-009-0434-z.

Nash, M. P., and Panfilov, A. (2004). Electromechanical model of excitable tissue to study reentrant cardiac arrhythmias. *Prog. Biophys. Mol. Biol.* 85, 501–522. doi:10.1016/J.PBIOMOLBIO.2004.01.016.
